# L-Type calcium channel blockade reduces network activity in human epileptic hypothalamic hamartoma tissue

**DOI:** 10.1111/j.1528-1167.2010.02942.x

**Published:** 2011-01-26

**Authors:** Kristina A Simeone, Shivkumar Sabesan, Do Young Kim, John F Kerrigan, Jong M Rho, Timothy A Simeone

**Affiliations:** * Divisions of Neurology and Pediatric Neurology, Barrow Neurological Institute, St. Joseph’s Hospital and Medical CenterPhoenix, Arizona, U.S.A.; † Department of Pharmacology, Creighton UniversityOmaha, Nebraska, U.S.A.

**Keywords:** Epilepsy, High-frequency oscillations, Nifedipine, Multielectrode array, Gelastic seizures, Hypothalamus

## Abstract

**Purpose:**

Human hypothalamic hamartomas (HHs) are associated with gelastic seizures, intrinsically epileptogenic, and notoriously refractory to medical therapy. We previously reported that the L-type calcium channel antagonist nifedipine blocks spontaneous firing and γ-aminobutyric acid (GABA)_A_–induced depolarization of single cells in HH tissue slices. In this study, we examined whether blocking L-type calcium channels attenuates emergent activity of HH neuronal networks.

**Methods:**

A high-density multielectrode array was used to record extracellular signals from surgically resected HH tissue slices. High-frequency oscillations (HFOs, ripples and fast ripples), field potentials, and multiunit activity (MUA) were studied (1) under normal and provoked [4-aminopyridine (4-AP)] conditions; and (2) following nifedipine treatment.

**Key Findings:**

Spontaneous activity occurred during normal artificial cerebrospinal fluid (aCSF) conditions. Nifedipine reduced the total number and duration of HFOs, abolished the association of HFOs with field potentials, and increased the inter-HFO burst intervals. Notably, the number of active regions was decreased by 45 ± 9% (mean ± SEM) after nifedipine treatment. When considering electrodes that detected activity, nifedipine increased MUA in 58% of electrodes and reduced the number of field potentials in 67% of electrodes. Provocation with 4-AP increased the number of events and, as the number of electrodes that detected activity increased 248 ± 62%, promoted tissue-wide propagation of activity. During provocation with 4-AP, nifedipine effectively reduced HFOs, the association of HFOs with field potentials, field potentials, MUA, and the number of active regions, and limited propagation.

**Significance:**

This is the first study to report (1) the presence of HFOs in human subcortical epileptic brain tissue in vitro; (2) the modulation of “pathologic” high-frequency oscillations (i.e., fast ripples) in human epileptic tissue by L-type calcium channel blockers; and (3) the modulation of *network* physiology and synchrony of emergent activity in human epileptic tissue following blockade of L-type calcium channels. Attenuation of activity in HH tissue during normal and provoked conditions supports a potential therapeutic usefulness of L-type calcium channel blockers in epileptic patients with HH.

Hypothalamic hamartomas (HHs) are intrinsically epileptogenic and are the origin of the characteristic medically refractory gelastic seizures (Munari et al., 1995; Arroyo et al., 1997; Kuzniecky et al., 1997). HH tissue is composed of small-neuron clusters that are interspersed with large neurons (Coons et al., 2007). Although small γ-aminobutyric acid (GABA)ergic neurons (10–16 μm) spontaneously fire action potentials (Wu et al., 2005; Kim do et al., 2008), the large neurons (20–28 μm and often embedded in a GABA-rich neuropil) are quiescent at resting membrane potential and depolarize in response to GABA_A_-receptor activation (Kim do et al., 2008; Kim et al., 2009; We et al., 2008). Serial reconstruction studies traced axonal projections from small neurons to symmetric (putative inhibitory) terminal synapses onto the soma of large neurons (Beggs et al., 2008), further supporting our current working hypothesis of HH tissue epileptogenicity (see Fenoglio et al., 2007): Clusters of small, spontaneously firing neurons release GABA onto large neurons and activate GABA_A_ receptors, inducing depolarization and action potential firing of large neurons. This hypothesis accounts for a hyperexcitable (and potentially epileptogenic) mechanism that is initiated by an intrinsic pacemaker, that is, the spontaneous firing of small neurons.

Blockade of L-type calcium channels with nifedipine abolishes GABA depolarization in 90% of large neurons and attenuates the spontaneous firing of small neurons, thereby dampening both modes of excitability within HH tissue (Kim do et al., 2008). However, whether these individual neuronal types collectively generate an epileptic network and whether network activity is attenuated after blocking L-type calcium channels remain unknown. A critical component of translational neuropharmacology research lies in understanding how the emergent properties of neuronal networks respond to pharmacotherapies (Faingold, 2004). Therefore, the goal of the current study was to examine the network activity of HH tissue in vitro using a multielectrode array. This study is significant because it bridges the gap between previously reported single-cell pharmacology and potential translational use of L-type calcium channel blockers as an anticonvulsant treatment. Specifically, we tested two hypotheses: (1) HH tissue is a spontaneously active neuronal network capable of generating epileptiform-like discharges; and (2) pharmacologic blockade of L-type calcium channels reduces epileptiform-like activity in HH tissue slices under normal and provoked conditions.

## Materials and Methods

### Clinical profile of patients

Tissue samples were obtained from 16 patients (10 female and 6 male) undergoing surgical resection at the Barrow Neurological Institute between March 2006 and February 2008. The mean age was 7.8 years (range 1.1–27.8 years). All patients had treatment-resistant epilepsy and had onset of epilepsy at 12 months of age or earlier, including eight (50%) with onset of epilepsy <1 month of age. At the time of surgery, 14 patients had multiple daily seizures, and the remaining 2 patients had weekly seizures. Eight patients (50%) had only gelastic seizures (mean age 4.8 years), whereas the remainder had multiple seizure types at the time of surgery (mean age 10.8 years). All patients had gelastic seizures at some point during their clinical course. Eight patients were taking more than one antiepileptic drug (AED) at surgery (Table S1).

According to the classification system proposed by Delalande and Fohlen (2003), one patient had a type 1 lesion, 10 type II, 2 type III, and 3 type IV. The mean HH lesion volume for all cases was 4.5 cc (range 0.2–20.3 cc). One patient had Pallister-Hall Syndrome and one had a prior history of HH treatment with gamma knife radiosurgery. Surgical resection of HH tissue was accomplished with the transcallosal, interforniceal approach in seven patients, and the transventricular endoscopic approach in nine patients. Pathology confirmed the diagnosis of HH in all cases. Informed consent was obtained from all patients, for the use of resected tissue for research purposes and for prospective database entry, in accordance with the Declaration of Helsinki; Title 45, U.S. Code of Federal Regulations, Part 46, Protection of Human Subjects; and National Institutes of Health (NIH) guidelines. Protocols were approved by the Institutional Review Board of the Barrow Neurological Institute and St. Joseph’s Hospital and Medical Center, Phoenix, Arizona.

### Slice preparation and multielectrode recordings

Human HH tissue was collected immediately after surgical resections. In the operating room, tissue specimens (ranging from 2 mm^3^ to 1 cc) were immediately transferred to 4°C oxygenated (95% O_2_ and 5% CO_2_) artificial cerebrospinal fluid (aCSF; composition in mm: 124 NaCl, 1.3 MgSO_4_, 3 KCl, 1.25 NaH_2_PO_4_, 26 NaHCO_3_, 2.4 CaCl_2_, and 10 d-glucose; pH: 7.4) and taken to the laboratory (within 5 min from the operating room). Specimens were sectioned (350–400 μm) and allowed to recover for 1 h in oxygenated aCSF at 32°C. Sections were transferred to room temperature, and aCSF bubbled with 95% O_2_ and 5% CO_2_. Sections were arbitrarily aligned over an entire 1 mm^2^ 64-planar electrode array and inserted into the MED64 connector (Alpha Med Systems, Osaka, Japan). The entire apparatus was placed in a thermoregulated CO_2_ micro-incubator (32°C) modified to allow perfusion lines and a line carrying humidified 95% O_2_/5% CO_2_ air. The slice was perfused (1 ml/min) with in-line prewarmed (32°C) oxygenated aCSF to ensure temperature control and allowed to acclimate for 1 h. Data were acquired in a continuous, gap-free mode at a sampling rate of 20 kHz with a bandpass width of 0.1 Hz–10 kHz using Conductor 3.0 software (Alpha Med Systems). Pharmacologic agents were dissolved in aCSF: 50 μm nonselective potassium channel blocker 4-aminopyridine (4-AP) and 100 μm L-type calcium channel inhibitor nifedipine (due to the scarcity of the tissue one dose was chosen for each drug). Drugs were purchased from Sigma-Aldrich (St. Louis, MO, U.S.A.). Spontaneous events were recorded in 5-min epochs. Multiple epochs were recorded at baseline, during each 40-min drug application and during the washout periods. Initial analyses of HH tissue sections from five cases indicated that baseline activity during 5-min epochs did not differ throughout the recording session.

One caveat the authors struggled with is the lack of appropriate control tissue. Due to the well-defined boundaries (see Kerrigan et al., 2005), surgical technique, and critical location, normal hypothalamic tissue is not resected with HH lesions. The origin of HH cells remains unknown. Rodent hypothalamus was considered for control tissue. As a region with several variegated nuclei, each with unique protein expression patterns, function, and electrophysiological responses, one potential candidate region was the spontaneously firing suprachiasmatic nucleus (SCN). However, in contrast to HH tissue, application of either 4-AP or L-type calcium channel antagonists to SCN neurons does not influence the firing rate of multiunit activity (MUA; Bouskila & Dudek, 1995; Walsh et al., 1995; Kononenko & Dudek, 2006; Kononenko et al., 2008). Therefore, the present data are generated only from human epileptic HH tissue. For an internal comparison, we assessed activity under normal aCSF conditions and after provocation.

### Data analyses and statistical methods

Power spectrum analysis was performed on the unfiltered data to detect high-frequency activity. Welch periodogram, a statistically rigorous estimator of power spectrum from field potential data, was applied to the data. Per electrode, we estimated the power spectrum using a nonoverlapping 250-ms sliding window. It is important to note that band-pass filtering of transients or nonsinusoidal oscillations may lead to spurious oscillations that are similar to real oscillatory discharges. This can lead to false detection of high-frequency oscillations (HFOs). Therefore, the unfiltered and filtered data were carefully reviewed visually to confirm the results of the power spectrum analysis.

Raw traces from each electrode were imported into Spike2 software (Cambridge Electronics Design, Cambridge, United Kingdom) and HFOs (i.e., ripples and fast ripples), field potentials, and MUA were analyzed. Main line hum was removed with a 60-Hz notch filter. Field potentials were identified after traces were subjected to an infinite impulse response (IIR) 50-Hz low-pass second order Bessel filter and down-sampled to 500 Hz. Threshold detection for field potentials was set at three times the standard deviation of the root mean squared value of the signal (SD-RMS). To detect MUA, raw traces were passed through a second-order finite impulse response (FIR) band-pass filter with a bandwidth of 500–3,000 Hz. The automated threshold for MUA detection was set at six times the SD-RMS. Burst analyses were performed to determine the characteristics of MUA bursts. Identification of a MUA burst required at least three spikes occurring within a 1-s interspike interval. Raw recordings were passed through an FIR 100–175 Hz band-pass filter to detect ripples (−3 dB points = 80 Hz and 200 Hz; 1,319 filter coefficients) or a 200–600 Hz band-pass filter to detect fast ripples (−3 dB point = 180 Hz and 619 Hz; 1,319 filter coefficients). The threshold detection of the troughs in each ripple and fast ripple were set at three times the SD-RMS. To determine the characteristics of these HFOs, burst analyses were conducted on each recording. Identification of a ripple burst required at least three consecutive spikes with a maximum interspike interval of 30 ms. Fast ripple bursts required at least three consecutive spikes and no more than a 6-ms interspike interval. Spatiotemporal propagation of synchronous events was determined with averaged waveforms. The electrode with the largest events was used to time stamp events. Averaging of all waveforms for each individual electrode was triggered by the time-stamped events. For each type of activity, percent change in activity compared to baseline was calculated for each electrode after drug treatment and analyzed using an unpaired *t-*test unless otherwise noted. Data are expressed as mean ± SEM unless otherwise noted.

## Results

### Spontaneous and GABA_A_ receptor–mediated network activity in HH tissue slices

**Figure 1:**
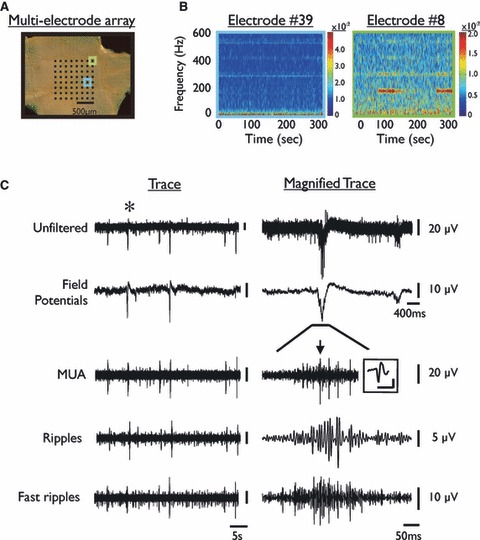
HH tissue generates spontaneous network discharges in vitro*.* (**A**) A digital image of an HH tissue section on a 64-electrode array. The 50-μm^2^ electrodes are positioned in an 8 × 8 grid with a 150-μm interpolar distance. (**B**) Power spectrum analysis over time for electrode 39 and 8 (electrodes are outlined in **A** with blue and green squares, respectively). The color bar on the right denotes the power at a particular frequency with high values of power represented in red and low ones in blue. Note that the presence of HFOs in electrode 8 and absence in electrode 39 illustrate local differences in HFO representation. (**C**) *Left:* Band-pass filtering of raw traces indicates the presence of field potentials, multiunit activity (MUA, 500–3,000 Hz), ripples (100–175 Hz), and fast ripples (200–600 Hz). *Right:* Expanded time scale of the event demarked with an asterisk. *Lower three traces:* Further time scale expansion to illustrate MUA, ripples, and fast ripples associated with the field potential. Single-unit waveforms were sorted (an example action potential denoted by the arrow is depicted in the insert; calibration bars of insert: 2 ms and 20 μV) and summated as MUA.

Using a high-density electrode array (Fig. 1A), spontaneous epileptiform-like activity, HFOs, field potentials, and MUA were detected throughout HH tissue slices during the course of each experiment (Fig. 1B). Epileptiform-like events similar to those reported in Taylor’s type focal cortical dysplasia (i.e., prolonged field potentials with superimposed MUA) and in human neocortical epileptic tissue (i.e., spontaneous field potentials >2 s duration; Köhling et al., 2000; D’Antuono et al., 2004) were apparent under normal aCSF conditions in HH slices. In addition, power spectrum analyses and further band-pass filtering revealed the presence of HFOs including ripples (100–200 Hz) and fast ripples (200–600 Hz; Fig. 1B). HFOs, specifically fast ripples, and their association with field potentials is a potential biomarker of epileptogenic tissue (Engel et al., 2009). MUA and HFOs occurred in association with field potentials and also independently of field potentials (Fig. 1C). Field potential discharges varied in amplitude (17–200 μV), duration (100 ms–12 s), rate of incidence detected by each electrode (13.4 ± 7.9 events/epoch), and distribution throughout the tissue. Field potentials rarely occurred synchronously. Under normal aCSF conditions, 1.6% of fast ripples and 1.4% of ripples were associated with field potentials. The frequency of MUA ranged from 0.01–2.7 Hz and occurred in regular, irregular, and bursting patterns similar to those observed during whole-cell patch clamp recordings of small HH neurons (see Kim do et al., 2008).

**Figure 2:**
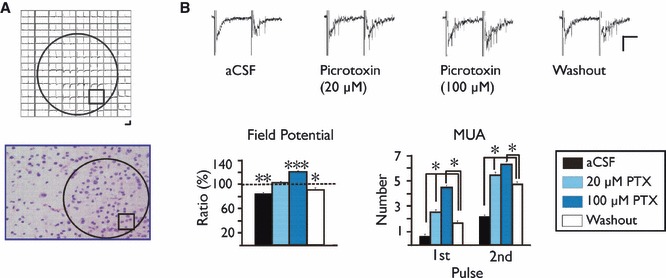
GABA-mediated neurotransmission. (**A**) Representative screen shot of 64 raw traces during paired-pulse stimulations in an HH tissue slice (the stimulation electrode is indicated by the black square). The propagation spread of MUA and field potential responses evoked in local neuronal populations resembles neuronal clusters (circled) apparent in HH tissue following hematoxylin and eosin staining (the 50-μm square is equivalent to the stimulation electrode). (**B**) Representative stimulation traces and quantification of paired-pulse ratios (the amplitude of the field potential response after the second pulse/that of the first) and the number of multi units (MUA) under normal conditions and following application of pictroxin (PTX). Horizontal calibration bars (**A**): 50 ms, (**B**) 25 ms. Vertical calibration bars (**A**): 100 μV, (**B**) 25 μV. Asterisks indicate statistical difference from 100% (paired-pulse ratio) or from aCSF conditions (MUA) *p < 0.05; **p < 0.01; ***p < 0.001.

GABA_A_-receptor activation hyperpolarizes small HH neurons and depolarizes large neurons (Kim do et al., 2008). To determine the role of GABA_A_ receptors in evoked transmission, we examined short-term plasticity within HH slices using a paired-pulse paradigm (50-ms interval, 200-μA biphasic stimulation; Fig. 2). MUA and field potentials were detected by local surrounding electrodes as far as 500 μm from the stimulation site with a spatial configuration resembling the neuroanatomic arrangement of small neuron clusters (Fig. 2A). Under normal aCSF conditions, there was a 5–20% paired-pulse depression and a fourfold increase in MUA with the second pulse (Fig. 2B). Blockade of GABA_A_ receptors with picrotoxin (20–100 μm) converted paired-pulse depression of the field potential to facilitation and increased evoked MUA. These results are consistent with a strong recurrent inhibitory network within small neuron clusters.

As anticipated, GABAergic modulation differentially influenced spontaneous emergent network activity. Following application of GABA_A_-receptor agonist muscimol (30 μm), MUA and field potentials were reduced in approximately half of the electrodes examined and were either increased or did not change in the remaining electrodes (Table S2). Blockade of receptors with picrotoxin (100 μm) increased network activity in approximately half of the electrodes, while either reducing or not influencing activity in the remaining electrodes (Table S2).

### L-Type calcium channel blockade modulates HFOs, field potentials, and MUA under normal aCSF conditions

**Figure 3:**
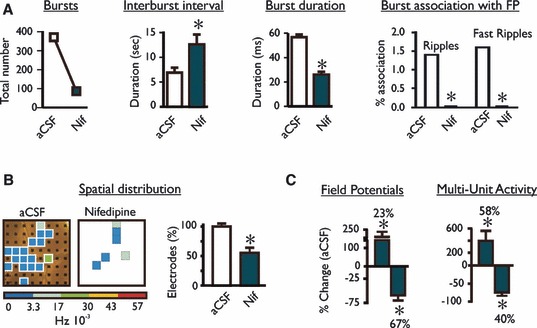
Nifedipine modulates spontaneous activity under normal aCSF conditions. (**A**) Blockade of L-type calcium channel attenuated ripple bursts and prevented the association of fast ripples and ripples with field potentials. (**B**) Representative spatial distribution of FP incidence under aCSF conditions and after nifedipine application. Bar graph depicts the total number of electrodes that detected activity under each condition; data are expressed as the mean percent ± standard error of the mean (SEM) relative to aCSF. (**C**) Bar graphs reflect the change in the number of field potentials and MUA after nifedipine treatment. Data are expressed as percent increase or decrease relative to baseline (aCSF) conditions. The bars below 0 indicate electrodes that detected less activity after nifedipine treatment. The bars above 0 reflect electrodes that detected increased activity after nifedipine application. The percent of electrodes that exhibited the respective change in activity relative to the total number of electrodes examined is displayed at the end of each bar. Data are expressed as the mean ± SEM. n = 9 slices, 6 cases; *significantly differs from aCSF conditions, p < 0.05.

We previously reported that blockade of L-type calcium channels suppresses the spontaneous firing of small neurons and the GABA-induced depolarization of large neurons (Kim do et al., 2008). Therefore, we hypothesized that an L-type calcium channel antagonist would reduce emergent network activity in HH tissue sections. Application of nifedipine reduced the total number of ripple bursts by 76% (from 371 to 87), significantly increased the interburst interval (6.9 ± 1.0 to12.6 ± 2.0 s, p < 0.05), reduced the burst duration (56.8 ± 2.2 vs. 26.0 ± 2.3 ms, p < 0.0001), and abolished the association of both fast ripples and ripples with field potentials (FPs; χ^2^ = 104.03, p < 0.001; Fig. 3A). In addition, application of the L-type calcium channel blocker reduced the number of regions exhibiting activity (i.e., the number of electrodes that detected activity) by 45 ± 9% (p < 0.005; Fig. 3B) and significantly attenuated the incidence of field potentials in a majority of electrodes (89 of 132) and of MUA in 19 of 48 electrodes (Fig. 3C). Interestingly, nifedipine increased the number of field potentials in a minority of electrodes (31 of 132) and MUA in a majority of electrodes (28 of 48; Fig. 3C). The remaining electrodes did not detect a change in activity. These data support the notion that treatment with an L-type calcium channel antagonist attenuates normal network activity in HH tissue sections under aCSF conditions.

### Blockade of L-type calcium channels reduces provoked activity in HH tissue slices

Our initial observations of HH tissue under aCSF conditions suggest inherent epileptiform-like activity. To determine whether the network elements of the pathologic tissue are able to generate more complex, synchronized seizure-like activity, the tissue was provoked with 4-AP (a nonspecific potassium channel blocker). Enhanced extracellular potassium levels have been shown during seizures, and similar provocation can be induced in vitro by artificially increasing the extracellular potassium concentration or by applying 4-AP (Lux & Heinemann, 1978; Lux et al., 1986; Avoli et al., 1999; Kivi et al., 2000; Dzhala & Staley, 2003; D’Antuono et al., 2004; Koch et al., 2005). We examined whether (1) provoking HH tissue with 4-AP increased excitability and elicited additional epileptiform-like discharges; and (2) nifedipine treatment reduced 4-AP provoked activity.

**Figure 4:**
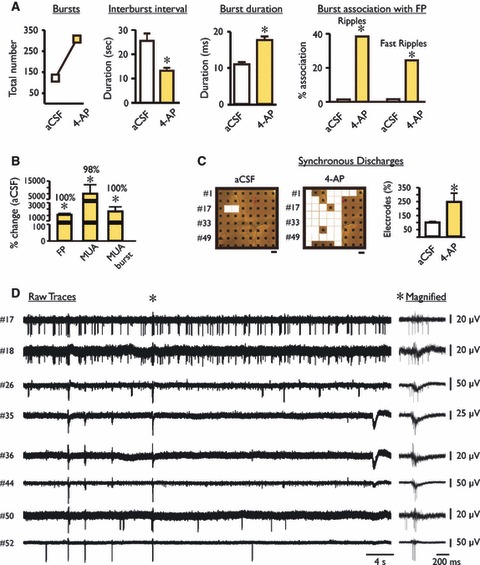
Application of the chemoconvulsant 4-aminopyridine (4-AP) significantly increases network discharges and synchronicity in HH slices. (**A**) Perfusion with 4-AP increased the total number of fast ripple bursts, reduced the interburst interval, increased burst duration, and significantly increased the association of fast ripples and ripples with field potentials (FP). (**B**) In addition, 4-AP substantially increased the number of FPs, multiunit activity (MUA), and the intra-MUA burst frequency in 100, 98, and 100% of electrodes examined, respectively (indicated above each bar). (**C**) Representative spatial distribution of increased synchronous discharges after 4-AP provocation. Electrodes 1, 17, 33, and 49 are labeled for reference. Bar graph depicts the total number of electrodes that detected activity under each condition; data are expressed as mean percent ± SEM relative to aCSF. (**D**) Traces from 8 of the 34 electrodes showing multiple synchronous events from 4-AP condition in C; the electrode number is indicated to the left. *Right*: Expanded time scale of event demarked with an asterisk depicts the unique types of activity that individual regions contribute to synchronous events. *Differs significantly from aCSF conditions, p < 0.05; n = 19 electrodes, 4 cases.

Application of 4-AP more than doubled the rate of incidence of fast ripple bursts as indicated by an increase in the total number of bursts (122 vs. 308), a significant reduction in the interburst interval (25.48 ± 3.06 vs. 13.22 ± 1.24 s, p < 0.005) and an increase in burst duration (10.99 ± 0.56 vs. 14.71 ± 0.96 ms, p < 0.05; Fig. 4A). Ripple burst characteristics were unaffected by 4-AP. In addition, fast ripples and ripples were more frequently associated with field potentials, increasing from 1.6% to 24.4% (χ^2^ = 22.11, p < 0.001) and from 1.4% to 38.4% (χ^2^ = 104.03, p < 0.001), respectively (Fig. 4A). There was significant increase in the incidence of field potential association with HFOs (0.3 ± 0.1 to 1.8 ± 0.3 events per min, p < 0.0005). Treatment with 4-AP recruited field potentials in an additional 7.0 ± 3.1 aCSF-silent electrodes per section (i.e., electrodes that did not detect activity under normal aCSF conditions, n = 6 cases). The number of field potential discharges increased substantially in 52 of 53 electrodes (1,000 ± 226%, Fig. 4B; 1 of 53 electrodes detected a decrease in field potentials). MUA burst firing has been identified as a sign of increased epileptogenicity in human epileptic neocortical preparations (Prince & Wong, 1981; Schwartzkroin et al., 1983; Avoli & Williamson, 1996). There was a significant increase in MUA and in intraburst MUA frequency in all electrodes examined after 4-AP application (22 of 22 electrodes, Fig. 4B). In addition, 5.8 ± 4.3 aCSF-silent electrodes (per section) detected MUA activity after 4-AP treatment.

Under baseline conditions, epileptiform-like events were rarely synchronous between electrodes; however, application of 4-AP promoted spatial synchronicity and propagation of events. The number of electrodes that detected activity increased significantly by 248 ± 62% (Fig. 4C). Notably, each region of the tissue provided a unique contribution to synchronous events, generating a field potential, MUA, HFOs, or a field potential associated with MUA and/or HFOs (Fig. 4D). Overall, provoking the tissue with 4-AP increased all types of neuronal activity and synchronicity in HH tissue sections.

**Figure 5:**
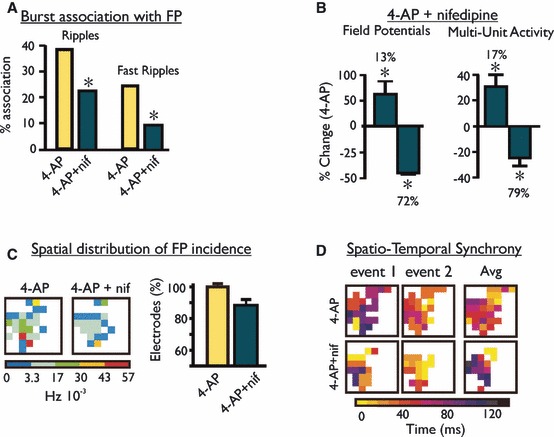
Blocking of L-type calcium channels attenuates provoked activity in HH tissue sections. (**A**) Bar graph depicts a significant reduction in the association of fast ripples and ripples with field potentials. Data are expressed as the percent of total HFOs associated with FPs. (**B**) Change in 4-AP-induced activity after application of 4-AP + nifedipine. Data are expressed as a percent change relative to activity during 4-AP treatment. The percent of electrodes that exhibited the respective change in activity relative to the total number of electrodes examined is displayed at the end of each bar. Data are expressed as the mean ± SEM. *Differs significantly from 4-AP activity, p < 0.05. (**C**) An example of spatial distribution of field potential incidence during 4-AP treatment and after application of 4-AP + nifedipine. Bar graph depicts the total number of electrodes that detected activity under each condition (for all slices). Data are expressed as a percent relative to 4-AP. (**D**) Two individual events and an average of all synchronous events derived from the same slice depict nifedipine inhibition of spatiotemporal propagation of 4-AP-induced synchronicity. Nif, nifedipine.

Blockade of L-type calcium channels attenuated 4-AP provocation of network activity in HH sections. Nifedipine substantially reduced the association of fast ripples and ripples with field potentials, by 64.3% and 42.7%, respectively (FPs; χ^2^ = 18.22, p < 0.001; χ^2^ = 10.13, p < 0.005, respectively; Fig. 5A). Other parameters of ripples and fast ripples were not affected by nifedipine (data not shown). Although a minority of electrodes detected an increase of field potentials (6 of 47 electrodes by 61 ± 28%) and MUA (4 of 24 electrodes by 30 ± 10.0%), the majority recorded a significant reduction of field potentials (34 of 47 electrodes) and MUA (19 of 24 electrodes, Fig. 5B; activity did not change in the remaining electrodes). In addition, there was a nonsignificant trend of fewer active regions after nifedipine treatment (p = 0.1; Fig. 5C). Nifedipine limited the extent of propagation of synchronous events throughout the tissue. Analysis of averaged waveforms of all events demonstrated that nifedipine did not alter the average generation site, but reduced the spatial representation and slowed the temporal spread of synchronous events (Fig. 5D, right panels). These data support the notion that treatment with an L-type calcium channel antagonist attenuates provoked network activity in HH tissue slices.

## Discussion

The current study is the first to report (1) the presence of ripples and fast ripples in human subcortical epileptic brain tissue in vitro; (2) spontaneous epileptiform-like discharges, field potentials, and MUA in human HH slices; and (3) the modulation of “pathologic” high-frequency oscillations (i.e., fast ripples), network physiology, and synchrony of emergent activity in human epileptic tissue by L-type calcium channel blockers.

The type(s) of network activity (i.e., HFOs, MUA, and/or field potentials) and conditions (i.e., under normal aCSF or provoked) that critically contribute(s) to the epileptogenicity of brain tissue has yet to be definitively established. HFOs, specifically fast ripples, are purportedly restricted to the seizure-onset zone, have been recorded during interictal events in the mesial temporal lobe and neocortex of patients with epilepsy, and appear at the same time as EEG spikes (Bragin et al., 1999; Engel et al., 2003; Urrestarazu et al., 2007; Schevon et al., 2009). Ripples occur in the early phase of seizure discharges and are located within and external to the seizure-onset zone (Allen et al., 1992; Urrestarazu et al., 2007). Although ripples are thought to be field potentials of summated inhibitory postsynaptic potentials, fast ripples are considered to reflect “field potentials of population spikes” from clusters of bursting neurons (Engel et al., 2009). Consistent with these findings, HH tissue is intrinsically epileptogenic, and the strong interplay between large excitatory “principal-like” neurons and clusters of local spontaneously firing small neurons creates a cytoarchitecture that is a prime environment to generate HFOs. Therefore, the presence of fast ripples and/or ripples and their association with field potentials may be a critical component of seizure genesis in HH tissue.

In addition to HFOs, spontaneous and induced field potentials also have been recorded in human epileptic tissues with single electrodes, and their occurrence has been associated with interictal and ictal EEG events (Schwartzkroin & Haglund, 1986). Synchronous sharp field potentials recorded in human epileptic neocortical slices arise from the initial activation of a small group of neurons firing tens of milliseconds before the field potential peak, and this initial local activation is followed by recruitment of additional neuronal groups with a spatial spread ranging from 200–750 μm (Köhling et al., 1998, 2000). The coherent activity of a local group of neurons is purported to initiate epileptiform discharges, and the recurrence of these field potentials has been implicated in reinforcing synchronous synaptic circuitry, thereby laying the foundation for subsequent ictal events (Traub et al., 1996; Staley & Dudek, 2006). The spontaneous field potentials in HH tissue under aCSF conditions and the increased number during provocation with 4-AP may indicate greater coherence of several groups of neurons reinforcing pathologic synaptic circuitry and reflect the tissue’s increased capacity to initiate epileptiform events.

The events recorded in each HH slice may reflect interictal activity. These events represent a small fraction of the network activity of the whole tissue in vivo. The constant spontaneous activity may transition into ictal activity and trigger extrahypothalamic propagation in response to provocation or an inciting event that excites the tissue resulting in subsequent change in potassium and calcium fluxes and initiation of calcium signaling cascades. When provoked in vitro, HH tissue activity increased by hundreds to >1,000% as did the synchronicity of events, suggesting that the cytoarchitecture of HH tissue is able to recruit neighboring networks that may support long-range propagation of seizures within HH tissue. When provoked in vivo, the summated and synchronized hyperexcitability may surpass seizure threshold and yield preictal/ictal activity. In patients, interictal spikes arise from synchronous activation of approximately 5–6 cm^2^ of cortex. Therefore, epileptiform-like activity (both interictal and ictal) in vitro may arise from the emergent orchestrated synchronicity of events throughout the tissue. We speculate that large projection neurons found in HH may relay the seizure activity to subcortical, cortical, and/or limbic regions resulting in the diverse phenotypic seizures types associated with HH (Kerrigan et al., 2005; Fenoglio et al., 2007).

Blockade of calcium channels has been reported to reduce epileptic activity in animal models of epilepsy and in human epileptic tissue (Köhling et al., 1998; Straub et al., 2000; Staley & Dudek, 2006). Reducing calcium entry may yield an anticonvulsant effect by weakening recurrent synapses, thereby reducing spike frequency and the probability of subsequent propagation of epileptiform-like events in vitro. Blockade of L-type calcium channels markedly reduced field potentials in HH tissue under normal aCSF and provoked conditions. In addition, this is the first study to report that blocking L-type calcium channels attenuated HFOs and abolished their association with field potentials in epileptic tissue. Therefore, preventing intracellular calcium flux through L-type calcium channels may limit synchronous depolarizations and reduce/uncouple HFOs from field potentials.

According to our working hypothesis, spontaneous action potential firing of small neurons drives epileptogenic activity of HH tissue (Fenoglio et al., 2007). MUA detected in the current study may reflect action potential firing of both small and large neurons with complex excitatory and inhibitory efferent projections. This is further supported by the conversion of paired-pulse depression (possibly due to small-neuron–mediated inhibition) to facilitation and increase in evoked MUA after blockade of GABA_A_ receptors with picrotoxin (20–100 μm; potentially attributable to the disinhibition of small neurons). One may speculate that the increased activity following nifedipine may reflect activity initiated by the ∼10% of large neurons that are insensitive to L-type calcium modulation. Nifedipine may also differentially affect the firing of clusters of small HH neurons, such that disinhibition of adjacent small neurons (via inhibitory collaterals) and of large HH neurons (via GABAergic input) occurs. The enhanced efficacy of nifedipine following provocation may be attributed to the increased efficacy of dihydropyridines in blocking L-type calcium channels under depolarized conditions (Bean, 1984).

In summary, the data from the current study suggest that nifedipine attenuates activity differentially under normal aCSF conditions (which may reduce seizure threshold) and under provoked conditions (i.e., during a seizure). Therefore, if critical epileptogenic elements include (1) the association of field potentials with HFOs under basal or provoked conditions; (2) the frequency of field potentials under basal or provoked conditions; (3) MUA during provoked conditions; and/or (4) propagation of events, then blockade of L-type calcium channels may be anticonvulsant in HH tissue and warrants consideration as an antiepileptic treatment for patients with HH.

HH patients typically exhibit seizures that are refractory to traditional anticonvulsant therapies. The identification of a novel molecular target may ultimately provide an effective pharmacologic option for HH patients with intractable epilepsy. The AED history of 14 of 16 patients indicates treatment with AEDs known to modulate both GABA_A_ receptors and calcium channels (Table S1; Simeone, 2010). The present study highlights the complexity of abnormal brain tissue and suggests that a more specific L-type calcium channel blocker may be efficacious in the treatment of seizures associated with HH despite the lack of a consistent effect in other types of epilepsies (see Kulak et al., 2004). Due to the peripheral side-effects and limited permeability of nifedipine, other L-type calcium channel blockers with more favorable pharmacokinetics and better central nervous system penetration such as amlodipine or nimodipine may be considered. Delineation of the mechanisms of seizure genesis operant in HH lesions and pharmacologic reduction of such activity will hopefully reveal facets of subcortical epileptogenesis that will also be relevant to other brain structures.

## Supporting Information

**Table S1.** Antiepileptic drug (AED) treatments of each patient.

**Table S2.** GABAergic modulation.
