# Supplementary material for: L-Type calcium channel blockade reduces network activity in human epileptic hypothalamic hamartoma tissue
Source: Epilepsia. 2011 Jan 26;52(3):531–40. doi: 10.1111/j.1528-1167.2010.02942.x (PMC3071288; doi:10.1111/j.1528-1167.2010.02942.x)
Supplement: Supplementary file 1 [file epi0052-0531-SD1.doc]

| **Supplemental Table 1. Anti-epileptic drug** (**AED**) **treatments of each patient.** | | | | | | |
| --- | --- | --- | --- | --- | --- | --- |
| Patient # | AED at surgery | | | Prior AED history | | |
|  | AEDs | Ca | GABA | AEDs | Ca | GABA |
| 1 | TPM, VPA | X | X | OXC | X | X |
| 2 | LEV | X | X | KLN |  | X |
| 3 | LEV | X | X | None |  |  |
| 4 | LEV, TPM | X | X | None |  |  |
| 5 | LEV, LTG, OXC | X | X | VPA | X | X |
| 6 | LEV | X | X | OXC, VPA | X | X |
| 7 | LEV | X | X | None |  |  |
| 8 | OXC | X | X | None |  |  |
| 9 | CBZ, LEV | X | X | TPM | X | X |
| 10 | OXC, TPM, PB | X | X | VPA | X | X |
| 11 | LTG, OXC | X | X | KLN,LEV,TPM,VPA | X | X |
| 12 | TPM | X | X | LEV | X | X |
| 13 | TPM, VPA | X | X | LEV | X | X |
| 14 | None |  |  | None |  |  |
| 15 | KLN |  | X | LTG | X |  |
| 16 | DPH, PGB, LEV, LTG | X | X | CBZ, FBM, GBP, PB, PRM, OXC, TPM, VPA, ZNS | X | X |

Modulation of calcium channels and/ or GABA receptors by each AED treatment strategy is noted. CBZ, carbamazepine; DPH, phenytoin; KLN, clonazepam; LEV, Levetiracetam; LTG, lamotrigine; PB, phenobarbital; PGB, pregabalin; OXC, oxcarbazepine; TPM, topiramate; VPA, valproic acid.

**Supplemental Table 2.** GABAergic modulation

| Drug | Activity | # of electrodes | Increased | # of electrodes | Decreased | No change |
| --- | --- | --- | --- | --- | --- | --- |
| Muscimol (30μM) | MUA | 8 of 65 | 1060±660% | 30 of 65 | 88±11% | 27 |
|  | FP | 39 of 98 | 140±67% | 36 of 98 | 93±7% | 23 |
| Picrotoxin (100μM) | MUA | 39 of 64 | 5370±2810% | 13 of 64 | 62±23% | 12 |
|  | FP | 43 of 86 | 222±114% | 27 of 86 | 72±12% | 16 |

FP, field potentials; MUA, multi-unit activity; n = 8 slices, 7 cases
